# Social interaction following prepubertal stress alters prefrontal gene expression associated with cell signalling and oligodendrocytes

**DOI:** 10.1038/s41398-022-02280-7

**Published:** 2022-12-16

**Authors:** Anna L. Moon, Nicholas E. Clifton, Natalie Wellard, Kerrie L. Thomas, Jeremy Hall, Nichola M. Brydges

**Affiliations:** 1 Neuroscience and Mental Health Research Institute, Cardiff University, Hadyn Ellis Building, Maindy Road, Cardiff, CF24 4HQ UK; 2 MRC Centre for Neuropsychiatric Genetics and Genomics, Cardiff University, Hadyn Ellis Building, Maindy Road, Cardiff, CF24 4HQ UK; 3 School of Biosciences, Cardiff University, Museum Avenue, Cardiff, CF10 3AX UK

**Keywords:** Molecular neuroscience, Physiology

## Abstract

Early-life adversity is associated with an increased risk of psychopathology, including mood disorders, later in life. Early-life stress affects several physiological systems, however, the exact mechanisms underlying pathological risk are not fully understood. This knowledge is crucial in developing appropriate therapeutic interventions. The prepubertal period is documented as a key developmental period for the maturation of the prefrontal cortex (PFC), a brain region involved in higher cognitive functions, including social function. In this study, we performed RNA sequencing on the PFC of adult rats who had experienced prepubertal stress (PPS) and controls to investigate the genome-wide consequences of this stress. PPS alters social behaviour in adulthood, therefore we also performed RNA sequencing on PPS and control rats following a social interaction test to determine social activity-dependent gene changes. At a baseline state (1 week following a social interaction test), no genes were differentially expressed in the PPS group. However, 1603 genes were differentially expressed in PPS rats compared to controls following a social interaction. These genes were enriched in biological pathways associated with cell signalling and axon myelination dynamics. Cell enrichment analysis showed these genes were associated with oligodendrocytes, and a comparison with an existing early-life stress sequencing dataset showed that pathways linked to oligodendrocyte morphology are impacted in a range of models of early-life stress in rodents. In conclusion, we identify pathways, including those involved in axon myelination, that are differentially activated in the adult in response to social stimulation following PPS. These differential responses may contribute to vulnerability to psychiatric pathology.

## Introduction

Stress experienced early in life has been consistently shown to have lasting negative consequences on mental health and has been associated with the later development of multiple types of psychiatric disorders [1–3]. Thus, unravelling the impact of stress on neurodevelopment is not only an important public health concern, but also gives insight into the mechanisms underlying psychopathology. This understanding of how early-life stress can shape neural and cognitive development is essential to inform both prevention and intervention strategies.

In humans, children with a history of childhood adversity have an increased risk of developing psychiatric disorders across diagnostic boundaries, including depression [4–6], schizophrenia [7] and bipolar disorder [8]. Childhood trauma has been associated with a range of physiological effects lasting into adulthood, including hypothalamic–pituitary–adrenal axis dysfunction [9], brain volume alterations [10] and hypervigilance to potential social threat [11].

Animal models have been extensively utilised to try to understand the underlying neurobiology that results in this vulnerability, however, the vast majority of studies focus on the early postnatal period [12]. It is also important to consider the later postweaning, peripubertal period, a sensitive developmental timepoint featuring maturation of the amygdala, hippocampus and prefrontal cortex (PFC), as well as brain-wide synaptic pruning [13]. Stress in this prepubertal period (PPS) has broad effects on behaviour with studies reporting, for example, increased anxiety in adulthood [14–18] and altered fear memory [19, 20]. We have previously found that PPS impairs social performance and increases expression of the social neuropeptide arginine vasopressin (AVP) in rats [21], consistent with work by other groups [22–24]. We also reported that the corticosterone response of female rats to a social test was significantly blunted in rats who had experienced PPS [25], reflecting findings in humans [26].

The PFC is highly involved in social function [27] and is likely to be especially sensitive to the effects of stress during the prepubertal phase, given its extended period of maturation [28]. The PFC has a high density of glucocorticoid receptors [29, 30], and its functioning is sensitive to stress in both humans and animals [31]. Coupled with significant maturation during early life, the PFC is predicted to be particularly vulnerable to the effects of early-life stress. Studies showing that adolescents exposed to early-life trauma demonstrate altered activation of the PFC when performing tasks involving emotional conflict [32] or negative affect [33] support this hypothesis. Early-life stress also affects the functional connectivity between the amygdala and the PFC [24, 32, 34], particularly during emotion regulation [34]. The neurobiological changes underlying these early-life stress-induced alterations are not well understood. The few studies evaluating gene expression changes following PPS have mostly concentrated on hypothesis-driven approaches focusing on genes involved in HPA axis regulation [35–37]. For example, we have previously reported gene expression changes in genes with a central role in HPA axis function, *Nr3c1* and *Avpr1a*, in the PFC of female rats following PPS [17, 21]. To fully evaluate the undoubtedly complex effects of PPS on gene expression, a genome-wide assessment of transcriptomic variation is necessary.

In this study, we aimed to capture genome-wide gene expression changes in the PFC of female rats exposed to PPS. As our previous study revealed changes in social behaviour following PPS [21], we hypothesised that stress during this time period would have significant effects on genes expressed during social function in adulthood. Thus, we predicted that while there may be changes in the gene expression profiles of PPS rats at a baseline state (1 week following a social interaction), these would be more pronounced directly following a social test. Due to the greater vulnerability of females to stress-related pathophysiology [38], and as we have previously observed social behaviour changes and expression changes specific to female rats [20, 25], we focused on female rats in this study.

## Methods

### Subjects

Female Lister Hooded rats were bred from adult pairs (Charles River) in house at Cardiff University and housed in same sex cages (32 × 50 × 21 cm) with littermates. Animals were housed on 12:12 h light–dark cycles (light phase 8 am–8 pm). All experiments were conducted in accordance with local ethics guidelines, the UK Home Office Animal Act 1986 and the European Communities Council Directive (1986/609/EEC). Twenty-four rats were used for social testing and tissue collection for RNA sequencing. The behavioural results from this cohort of rats has previously been published [21]. We focused on females as women are twice as likely to develop stress-associated pathology.

### Prepubertal stress

**Fig. 1:**
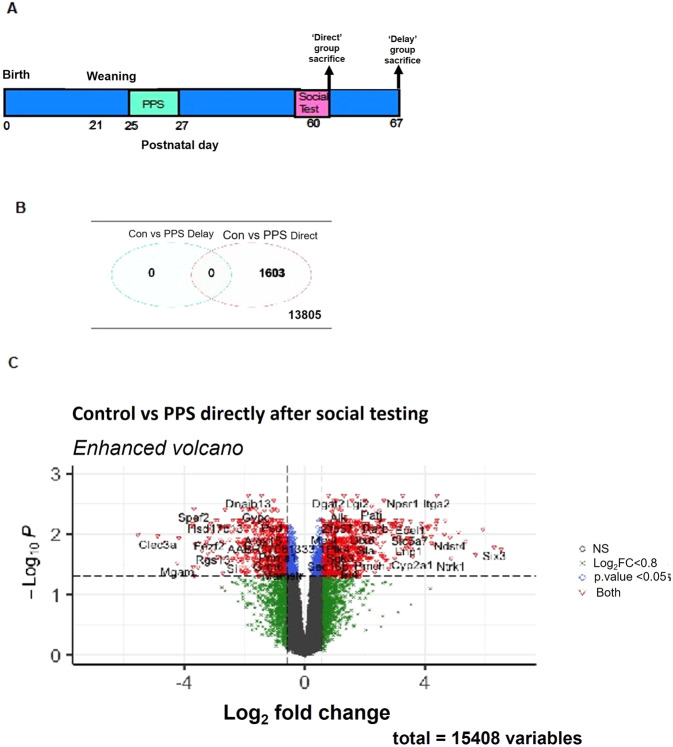
Experimental timeline and differentially expressed genes. **A** Timeline of the experiment. **B** Venn Diagram displaying DEGs between CON vs PPS animals directly following a social test (Con vs PPS Direct) and in a baseline state, 1 week after the social test (Con vs PPS Delay) following *limma* analysis. No DEGs were seen in the Delay (baseline) group. 1603 DEGs were observed in the Direct group, whilst 13805 genes did not differ in expression. **C** Enhanced Volcano Plot demonstrating DEGs in the Direct group (Con vs PPS). All genes above the dotted line were differentially expressed, green points represent log2FoldChange, blue represent genes <0.05 and red points represent those genes that meet both criteria. Genes were considered differentially expressed if *P* < 0.05 following Bonferroni correction. DEGs differentially expressed genes, CON control, PPS prepubertal stress.

PPS was given to half of the litters on the postnatal day (PND) 25–27 following weaning on PND21 (Fig. 1A). Control litters were left undisturbed until PND60. This protocol has been described previously [15, 18, 21]. Briefly, on PND25, animals were given a 10 min swim stress in an opaque swimming tank (12 L capacity with 6 L of 25 + /− 1 °C water). On PND26, animals were given three sessions of 30-min restraint stress in plastic restraint tubes (15 cm × 5 cm diameter) with 30 min intervals between sessions. Lastly on PND27, rats were given 3 sessions of 30 min elevated platform exposures (15 × 15 cm, 115 cm high) with an hour interval between sessions (Fig. 1A). All stressors were conducted in a designated room, away from the holding room. Following these stressors, animals were returned to their home cages and left undisturbed until adulthood (PND60-70), apart from regular cage cleaning. Litters were randomly allocated to experimental groups based on the order of birth.

### Social testing

Social testing was conducted in adulthood in all rats: rats subject to PPS and controls. This procedure has also been described previously [21]. Three hours before testing, animals were single housed in their holding room to increase the desire for social contact, before being transferred to the testing room one hour before the test session for habituation to the novel testing environment. Testing took place in a novel clear acrylic arena (65 cm × 65 cm × 40 cm high) placed in the centre of a room. The social test consisted of placing two animals into opposite sides of the arena, facing the wall. Animals had not met before but were the same sex and from the same group (control (CON) or PPS). The two rats were then allowed to freely interact for 15 min, before being returned to their home cages. The area was cleaned thoroughly with 70% ethanol between animals. The experimenter was blind to the experimental group during social testing.

### Tissue and plasma collection

Twenty minutes after social testing, half of the animals (one from each socially tested pair) were sacrificed using a rising concentration of CO_2_. Brains were extracted, the prefrontal cortex (PFC) microdissected and snap-frozen on dry ice. This group is referred to as the ‘Direct’ timepoint throughout this study, and was used to compare differences in gene expression evoked by social testing between CON and PPS animals. This time point (35 min post onset of the social test) was selected as peak for the increase/decrease of social activity-evoked corticosterone [39, 40] and early downstream effects on gene expression. We acknowledge that sampling at other time points may reveal additional changes in expression, as different genes may display different expression profiles following social stimulation. The other half of the animals (one from each pair) were returned to their cages for 1 week, to allow any socially evoked gene expression changes to return to baseline, then sacrificed as above and are referred to as the ‘Delay’ timepoint (Fig. 1A). The Delay group were left for 1 week, as although the expression of most RNAs will return to baseline within a few hours, others can take days [41]. The Delay group provides an effective comparator group for isolating differences in socially evoked gene expression between the control and PPS rats from gene expression regulated by PPS and social interaction independently, and any non-specific differences (e.g., general arousal, novelty and locomotor activity) consequent to behavioural testing.

### RNA extraction and quality control

RNA was extracted from 24 PFC samples using a Qiagen RNeasy UCP Micro Kit according to the manufacturer’s instructions. An equal number of samples were used within each group (Con and PPS) and timepoint (Direct and Delay). Animals originated from 14 litters, and no more than two littermates were included in any one group to reduce the effects of pseudoreplication. RNA concentration was quantified using a Qubit^TM^ RNA High Sensitivity Assay Kit and Qubit 2.0^TM^ Fluorometer (Invitrogen, UK). RNA integrity and purity were quantified using a Bioanalyzer RNA 6000 Nano Assay (Agilent, UK) and run on a 2100 Bioanalyzer system (Agilent, UK). All samples had an RNA Integrity Number (RIN) greater than 9. Sample sizes were based on power calculations from previous studies using similar models and analyses [42].

### Library preparations and sequencing

cDNA libraries were created by poly(A) mRNA capture, using a KAPA mRNA Hyperprep Kit (Roche, Switzerland) according to the supplied protocol. 120 ng total RNA per sample was used as an input and KAPA Unique Dual-Indexed Adaptors were incorporated into each sample to allow for identification after sequencing. Following library amplification, libraries were run on a 2100 Bioanalyzer system using Agilent High Sensitivity DNA Kit to determine library purity and fragment size (average = 374 bp, SD = 10 bp). Three samples (1 from CON Delay, 1 from CON Direct and 1 from PPS Direct) were removed from the analysis as they did not form sufficient libraries. Library concentration was assessed by a QubitTM dsDNA High Sensitivity Assay Kit (Invitrogen, UK). Libraries were pooled together to achieve a final DNA molarity of 6.8 nM. Sequencing was performed on Illumina NovaSeq 6000 Next Generation Sequencer on an S2 flow cell with 100 bp paired-end reads at an average read depth of 75 million reads.

### RNA sequencing analysis pipeline

Trimmomatic [43] was used to remove adaptors and low-quality bases from reads, using default parameters. The quality of raw sequencing data was verified using FastQC analysis [44]. STAR [45] was used to map all reads to the rat reference genome (Rn6) and bamtools were utilised to mark duplicates and to check for equal ratios of reverse and forward counts [46]. Gene-level counts were generated using *featureCounts* [47]. Counts data was subjected to TMM normalisation before differential expression analysis using *limma* modelling [48, 49] implemented in R (version 4.0.3) to identify genes differentially expressed between CON and PPS animals at both the Delay and Direct timepoints. Limma-generated differential gene expression analysis models the variance for all genes in the genome in group comparisons, and resulting t-values, reflecting the effect size to standard error ratio, are reported in Supplementary Tables. Benjamini Hochberg (FDR) correction was used to adjust for multiple testing. Genes were considered differentially expressed genes (DEGs) if corrected *P* value <0.05. All data met the assumptions of statistical testing.

### Pathway analysis

Biological pathways overrepresented within the DEGs were investigated by gene ontology (GO) term enrichment analysis, using Fisher’s Exact Test. Gene sets defined by biological pathways were curated from the GO dataset, downloaded 4th March 2021. A custom background gene set was created using all tissue-expressed genes in the dataset (13,659 genes) to control for expression bias. Significant pathways were subject to refinement to determine the most specific pathways contributing to the effect, as described previously [50]. The same methods were applied to investigate phenotypes associated with our DEGs using the Phenotypes, Alleles & Disease Models project in MGI. Pathways were considered significant at *P* < 0.05 after Bonferroni correction.

### Cell-type enrichment and data comparisons

Brain region specific cell-type-specific lists of genes were obtained from an online repository (http://neuroexpresso.org), utilising the cortex region data. Genes were considered a cell-type-specific marker if the average expression was >8x the average background expression of all other cell types in that region [51]. The enrichment of our DEGs within these marker lists was determined using one-tailed Fisher’s Exact Tests using the GeneOverlap tool in R [52]. To observe whether our DEGs intersected with other gene sets of interest, including those from studies of early-life stress, we used the GeneOverlap tool as before. If necessary, mouse Ensembl gene IDs were converted to rat Ensembl IDs using the biomaRt tool implemented in R [53]. For early-life stress (ELS) stress gene set analysis, we used the nominal *P* value for Peña et al.’s [42] work as they did not observe any significant DEGs following correction for multiple comparisons. A custom background gene set was used to control for all brain-expressed genes.

## Results

### PPS animals show significant differential gene expression in the PFC directly following a social test when compared to controls

**Fig. 2:**
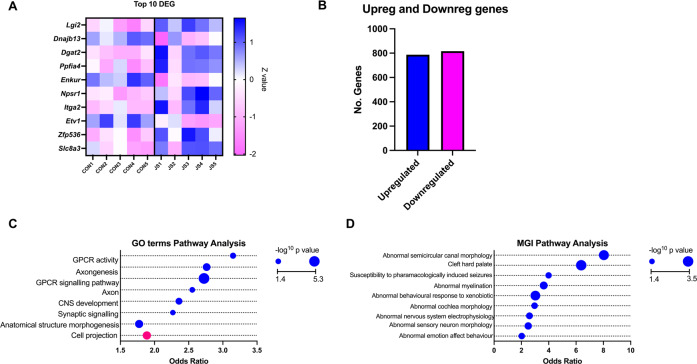
Downstream analysis of DEGs between CON vs PPS animals directly following a social test (Direct group). **A** Heatmap detailing the top 10 DEGs (as classified by adjusted *P* value) and their expression patterns: most of the top ten genes are upregulated in PPS animals compared to CON. Colour denotes Z value, blue indicates higher expression in comparison to average control expression. **B** Bar chart detailing the number of DEGs up- and downregulated. **C** Gene ontology terms enriched in DEGs, blue circles denote pathways from genes upregulated in PPS animals compared to CON, pink circles pathways from downregulated genes. **D** Phenotype analysis with terms from MGI’s Phenotypes, Alleles & Disease Models. Bonferroni *P* values form the circle size and blue circles denote pathways from upregulated genes. There were no significant terms from downregulated genes. DEGs differentially expressed genes, CON control, PPS prepubertal stress.

At the ‘Delay’ timepoint, no significant DEGs were found between the CON and PPS groups following correction for multiple testing (Supplementary Table 1). However, at the ‘Direct’ timepoint (20 min following a social test), 1603 DEGs were observed (Bonferroni *P*adj <0.05) in the PFC between CON and PPS rats (Fig. 1B and Supplementary Table 2). There were similar numbers of upregulated (816) and downregulated (787) genes (Figs. 1C and 2B). Many genes exhibited large changes in expression, with log2 fold changes ranging from −5.53 to +7.63 (Supplementary Table 2). Within the top ten DEGs were genes involved in calcium homoeostasis (*Enkur, Slc8a3*) and *Npsr1*, a gene that encodes neuropeptide S receptor 1—a member of the vasopression/oxytocin family of GPCRs (Fig. 2A).

### Differentially expressed genes were enriched for pathways pertaining to cell signalling mechanisms and phenotypes associated with the abnormal nervous system

Gene ontology analysis revealed 37 biological pathways enriched within the ‘Direct’ DEG gene set (Supplementary Table 3), which corresponded to 9 major pathways following refinement (Fig. 2C). Several of these implicate cell signalling, including GPCR signalling, second messenger signalling and synaptic signalling (Fig. 2C). This may reflect the activity-regulated brain processes induced by the social test, and suggests that PPS and CON animals responded to the social interaction test differently on a molecular level. Interestingly, pathways relating to the axon (‘axon’ and ‘axonogenesis’) were also significant.

We split ‘Direct’ DEGs into genes that were upregulated or downregulated in PPS animals in respect to CON, and tested again for pathway enrichment. All observed pathways were overrepresented among upregulated genes, apart from “cell projection” which was enriched in association for downregulated genes (Fig. 2C).

Pathway analysis using the MGI Phenotype database revealed 26 abnormal phenotypes enriched within DEGs between PPS and CON animals directly following a social test (Supplementary Table 4), which was refined to 10 independent terms (Fig. 2D). These included abnormal nervous system physiology and phenotypes involved in locomotion (Fig. 2D). DEGs were again split into up and downregulated genes and all significant pathways were enriched in upregulated genes.

### Oligodendrocyte-specific genes are enriched in PPS DEGs

**Fig. 3:**
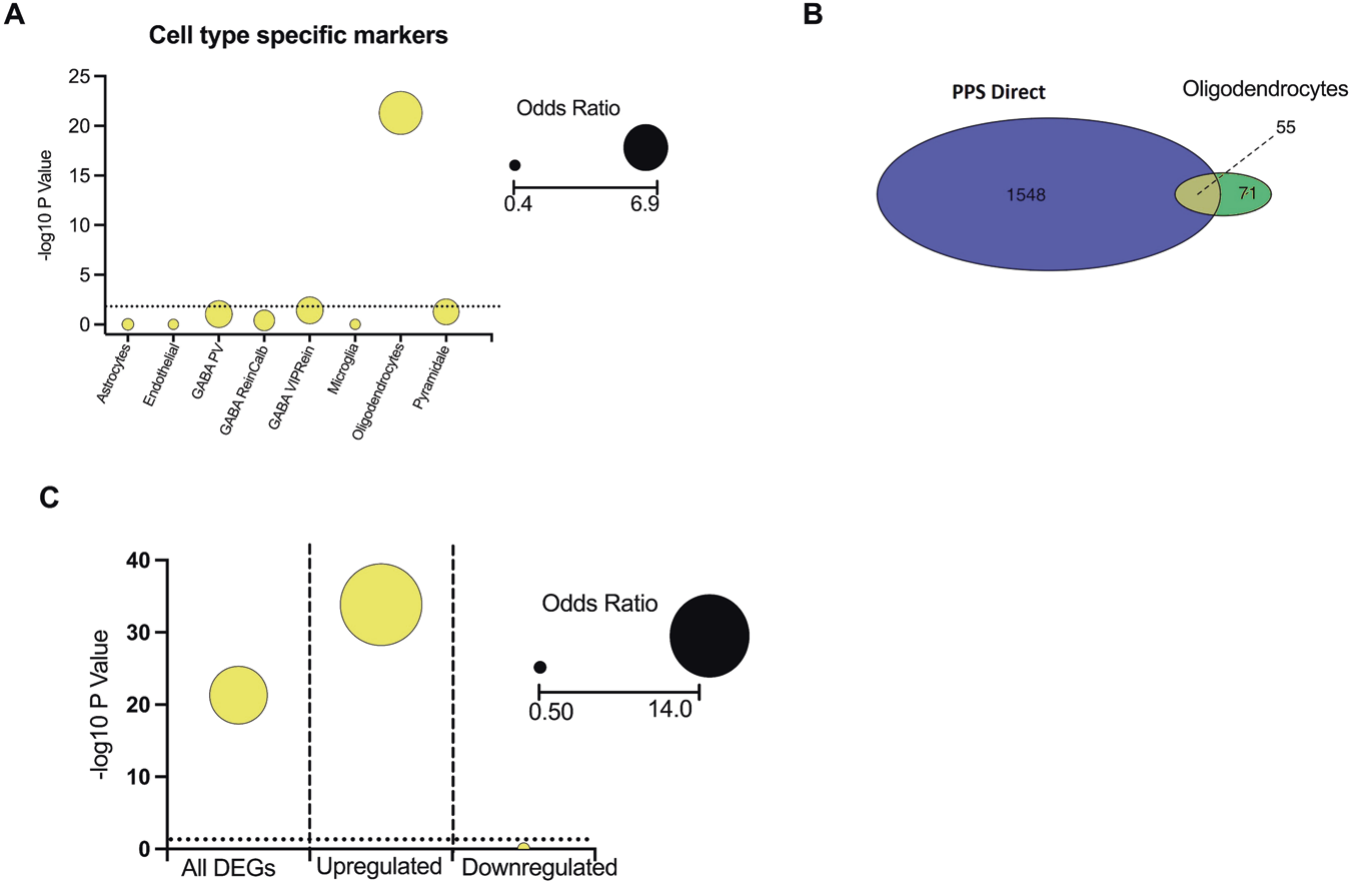
Cell-type enrichment analysis of DEGs between CON vs PPS animals directly following a social test (Direct group). **A** Cell-type-specific markers were compiled from a cortical cell-type-specific list and compared for enrichment within our DEGs. We demonstrate enrichment of oligodendrocyte-specific markers. The dotted line indicates the *P* value significance line, and the colour and size of the points demonstrate the odds ratio. **B** Venn diagram demonstrating the overlap between DEGs and oligodendrocyte-specific markers. **C** When DEGs were split into upregulated and downregulated genes, we see this signal comes solely from upregulated genes. DEGs differentially expressed genes, PPS prepubertal stress.

Next, we investigated whether a social test instigated different cell-type-specific transcriptional changes in PPS rats compared to controls. Curated cell-type-specific marker lists for cortical regions were extracted from a dataset of brain cell-type transcriptomes [50] and tested for overlap with our DEGs to observe any enrichment in a particular cell-type (Fig. 3A, B). There was a significant enrichment of oligodendrocyte-specific genes in our DEG dataset (OR = 6.9, *P* = 5.1 × 10^−22^), suggesting that oligodendrocytes may be a cell type particularly susceptible to PPS. Once again, when the dataset was split into up- and downregulated genes, it was found this enrichment was only evident in the upregulated genes (OR = 13.8, *P* = 1.5 × 10^−34^), but not the downregulated genes (OR = 0.3, *P* = 0.99) (Fig. 3C).

### Upregulated DEGs in PPS animals directly after a social test are enriched for association with glucocorticoid receptor binding sites

**Fig. 4:**
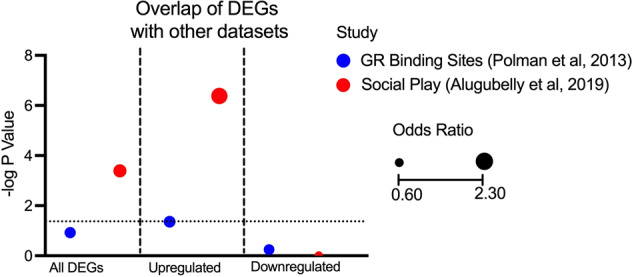
Enrichment of DEGs between CON vs PPS animals directly following a social test (Direct group) within all existing relevant datasets. Different datasets are plotted on the *x* axis with −log10 *P* value on the *y* axis indicating if there is significant overlap between each set and DEGs found in this study. Significant *P* values appear above the dotted line. The size of the points indicates the odds ratio. DEGs differentially expressed genes, CON control, PPS prepubertal stress.

Gene expression datasets from previous studies of stress regulation in cells and social play were investigated for overlap with our DEGs (Fig. 4). Polman et al. [54] conducted a ChIP-seq experiment in which they identified genomic binding sites of glucocorticoid receptors (GRs) in the rat hippocampus using a corticosterone assay [54]. Mapping of these sites to protein-coding genes resulted in 1549 genes validated as GR-binding sites, however, there was no significant gene overlap between these and our DEGs overall (*P* = 0.12). When the dataset was split into up and downregulated genes, we observed that upregulated genes were enriched for GR-binding sites (OR = 1.2, *P* = 0.044). However, it should be noted that GR-binding sites in the hippocampus may be different to sites in the prefrontal cortex, and a dataset of GR-binding sites in the PFC would be beneficial to completely answer this question.

### PPS animals have reduced gene expression associated with social play directly after a social test

**Fig. 5:**
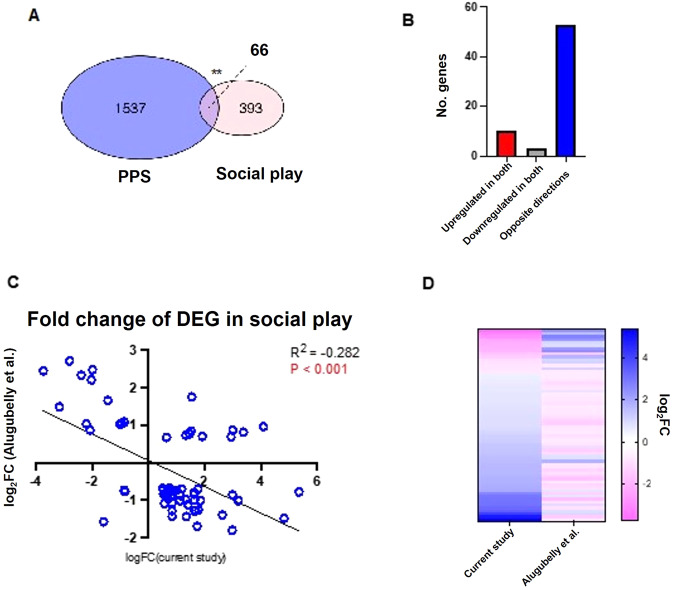
Overlap of differentially expressed genes with those arising from a social play study. **A** Venn diagram detailing the significant overlap between DEGs in the Direct group (CON vs PPS) and an amygdala social play dataset detailed in Alugubelly et al. [55]. **B** Bar chart showing that most overlapping genes are in opposite directions in both studies. **C** Fold changes of both datasets are significantly negatively correlated with one another. **D** Heatmap showing that expression of overlapping genes are in different directions, suggesting that genes upregulated during social play in the amygdala are downregulated in the PFC of PPS rats during a social interaction. DEGs differentially expressed genes, CON control, PPS prepubertal stress.

A study of DEGs in the amygdala of male rats following 15 min of social play compared to home cage controls [55] has previously reported a collection of 459 genes whose expression is altered following social play. There is a significant overlap between the DEGs altered following social play and the genes altered between CON and PPS in this study (OR = 1.5, *P* = 0.00231) (Figs. 4 and 5), which is again driven by the upregulated genes (OR = 2.3, *P* = 4.2 × 10^−7^). Furthermore, there is a significant negative correlation between the fold changes in each dataset (Pearson’s Correlation: *R*^2^ = −0.282, *P* < 0.001) (Fig. 5C), suggesting that genes typically increased following social play are decreased in PPS animals. While these experiments took place in different brain regions and sexes which may account for the differences, it may also suggest that PPS animals process the social test differently than CON animals.

### DEGs between CON and PPS rats directly after a social test correlate with previously reported DEGs from a combined early-life and adult stress paradigm

**Fig. 6:**
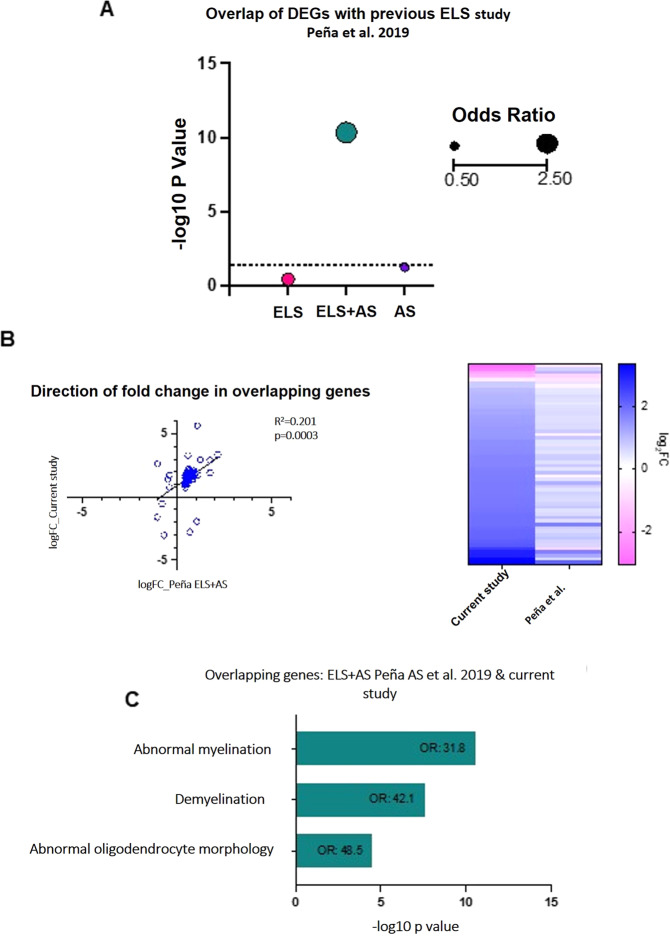
Overlap of DEGs between CON vs PPS animals directly following a social test (Direct group) with ELS DEG databases. **A** Enrichment of DEGs reported in Peña et al. [42] within our dataset demonstrates a significant overlap between our DEGs and those in the PFC of female mice exposed to ELS + AS. **B** Correlation analysis with DEGs in Peña et al. [42] reveals a significant positive correlation of fold changes, suggesting that genes are altered in the same direction, as shown in the heatmap. **C** MGI Phenotype pathway analysis on the overlapping genes from the current study and ELS + AS in Peña et al. [42] revealed phenotypes associated with dysregulated oligodendrocytes and abnormal myelin. DEGs differentially expressed genes, CON control, PPS prepubertal stress, ELS early-life stress, AS adult stress.

To our knowledge, no other study has performed RNA sequencing following PPS in preclinical models. However, Peña et al. [42] have performed extensive RNA sequencing on an ELS model and adult stress (AS) paradigms in various brain regions in mice [42]. Here, ELS was defined as maternal separation with limited nesting material between PND10-17. AS consisted of a ‘sub-threshold variable stress’ paradigm where mice experienced 100 random mild foot-shocks for 1 h, a 1-h tail suspension stress and a 1 h restraint stress over 3 consecutive days. We extracted the PFC data for female mice and compared the DEGs to those seen in our study. We did not observe a significant overlap following the ELS paradigm (OR = 0.8, *P* = 0.75) (Fig. 6A) or the AS paradigm (OR = 1.0, *P* = 0.5) in isolation (Fig. 6A). However, we see a highly significant overlap between DEGs in our current study and DEGs reported when ELS and AS was experienced in the same animal (Fig. 6A) (OR = 2.5, *P* = 4.5 × 10^−11^). Correlation analysis revealed a significant positive correlation between the fold changes in each dataset (*R*^2^ = 0.201, *P* = 0.0003) (Fig. 6B), showing that the gene variations were in the same direction (Fig. 6B).

### Pathway analysis on ELS studies reveals a shared overlap of genes associated with oligodendrocytes and myelination

The overlapping genes from our DEGs and Peña et al. [42] ELS + AS dataset were extracted, and pathway analysis was conducted using gene ontology analysis and MGI phenotype analysis. No GO terms were significant following correction, however, MGI phenotype analysis revealed that terms associated with abnormal myelin sheath morphology and oligodendrocyte morphology were common to both datasets (Fig. 6C). This suggests that despite differing models and timing of stress, they may have common effects on myelination and oligodendrocytes following early-life stress.

## Discussion

In this study, we report a genome-wide analysis of gene expression changes after PPS in the PFC of female rats, at a baseline state (1 week following a social test) and in direct response to a social test (20 min post test). We report the differential expression of 1603 genes induced by PPS directly after a social test. These genes were enriched in pathways associated with cell signalling mechanisms, axon dynamics and oligodendrocytes. These DEGs also overlap with other PFC datasets in mice, where stress is given both very early in life and in adulthood, suggesting that we have identified a set of genes that are consistently affected by stress across rodent species, timepoints and behavioural protocols. Despite similar numbers of upregulated and downregulated DEGs, it was seen that the upregulated genes drove the majority of these associations.

This study highlights the importance of examining both basal and activity-driven gene expression changes following early-life stress. While transcripts may be unaltered in baseline states, engagement in behavioural tasks or exposure to stress may reveal pronounced changes in gene regulation within an altered circuit. In the current study, the experience of a social test evoked significant gene expression changes in the PFC of rats exposed to PPS. The medial PFC is a central driver of social behaviour across species, supporting the selection of appropriate responses within a social environment through its widespread integration and interactions with numerous other brain regions and circuits [56–58]. With regards to social behaviour, medial PFC projections to the dorsal periaqueductal grey, raphe nucleus, nucleus accumbens, basolateral amygdala, hypothalamus, thalamus and hippocampus are known to be particularly important [56–58]. Future studies examining coordinated gene expression changes in these areas following social testing would be extremely informative. This activity-driven response reflects existing literature. We previously found that female rats given PPS displayed blunted corticosterone responses following a social test, within no changes at baseline [25]. Similarly in humans, early-life adversity results in exaggerated differences in HPA axis activity following exposure to social stress when compared to baseline [59, 60]. As stress is so closely tied to body homoeostasis and ability of the body to regulate hormone changes, it is important to consider how early-life stress may affect reactions to external events as well as at baseline to fully understand the consequences of early trauma on the underlying neurobiology and subsequent risk for disorder. One mechanism through which PPS could alter the effects of social interaction on the PFC transcriptome is via developmental programming. PPS increases levels of stress hormones, such as corticosterone. The PFC has a high density of corticosteroid receptors and is undergoing significant maturation during the prepubertal period [28–31]. Therefore, exposure to stress during this period could permanently alter the gene expression profile of the developing PFC, an effect which may only become apparent when gene expression is evoked through a behavioural challenge. Indeed, following a social test, we find that upregulated genes are enriched for GR-binding sites, and several other genes which interact with the GR system. For example, genes involved in axon myelination and oligodendrocyte morphology are altered, and these processes are known to be regulated by glucocorticoids [61].

Our previous study showed that PPS altered social behaviour in adulthood, with a decreased latency to initial contact and decreased duration of contact [21], as well as a blunted corticosterone response to social interaction in females [25]. This phenotype suggests that the underlying molecular pathways accompanying social interaction in PPS rats are altered. The current study supports this hypothesis with large gene expression changes seen directly after a social test in the PFC of PPS rats compared to controls. The pathways that are enriched in the DEG gene set include signalling pathways, such as synaptic and GPCR signalling, suggesting that the activity-driven pathways engaged by social interactions may be programmed differently. During the prepubertal period, the PFC undergoes significant maturation, and is highly stress-sensitive with high densities of GRs [30, 62]. Therefore, PPS may reprogramme how the PFC and its associated circuits (such as the hippocampus or amygdala) respond to a social interaction or a stressor, resulting in altered behaviour and widespread disruption of gene expression such as we report here. This is supported by previous literature. Structural changes are observed in the prefrontal cortex following a range of childhood adversities, and adults experiencing childhood maltreatment are more sensitive to social exclusion in a laboratory-based task, displaying associated increases in activity in the prefrontal cortex [63–65].

In this study, upregulated DEGs following PPS were enriched for GR-binding sites, suggesting that the stress response system may be over-activated during the social test. In support of this hypothesis, several studies have linked childhood adversity to abnormal HPA axis function during the Trier Social Stress Test [66].

One key finding of our study is the link between genes differentially expressed between PPS and controls following a social interaction and axon dynamics, myelination and oligodendrocytes. Several pathways implicated in axon morphology and function were enriched within our dataset (Fig. 2 and Supplementary Table 3), and oligodendrocyte-enriched cell markers were also identified as overrepresented within our DEGs. Furthermore, oligodendrocyte morphology was highlighted as a potential central mechanism that early-life stress may act upon regardless of type of stressor and timing (Fig. 6). Oligodendrocytes are responsible for the maturation, survival and integrity of myelinated axons, and axon myelination is crucial for the functioning of the nervous system and normal cognition [67]. Various oligodendrocytes abnormalities have been noted in depression, schizophrenia, and bipolar disorder and more specifically, depression has been shown to lead to reduced oligodendrocyte number in the PFC [68] and the downregulation of genes related to oligodendrocyte function [69, 70].

Notably, studies in adults with a history of childhood abuse have demonstrated axonal integrity disturbances [67] and furthermore in preclinical studies, maternal separation and limited nesting leads to reduced expression in proteins involved in axonal growth [71]. Brain imaging studies on children who experienced early-life neglect in Romanian orphanages have reported white matter alterations and reduced axonal integrity throughout the brain compared to children raised in a family setting [72, 73]. Interestingly, removing the children from an institution and placing them with foster families resulted in less severe white matter disturbances, highlighting the importance of intervention in early life [72, 73]. Furthermore, postmortem studies have found cell-type-specific changes in oligodendrocyte gene methylation and a global impairment in myelin-related transcription in depressed individuals who completed suicide and had a history of severe childhood abuse, an effect that was reflected in an animal model [74]. This suggests that oligodendrocytes may be a site of biological convergence following early-life stress, a hypothesis which should be followed up in further studies.

Our previous study reported increased AVP in rats following PPS and in humans with significant experience of childhood adversity [21]). In the present study, one of our most significantly differentially expressed genes was *Nspr1*, which encodes for neuropeptide S, part of the vasopression family, providing further evidence for the disruption of social peptides in this model. Also worthy of note is that *Avpr1a*, a receptor for AVP, is also significantly decreased in PPS rats (Supplementary Table 2, *P* = 0.023), replicating what we previously reported in a candidate gene-driven approach in a previous study [25]. AVP is an evolutionarily conserved neuropeptide that modulates social behaviour across species, and expression is also altered following maternal separation [75–77]. We found that altering AVP expression using an antagonist reverses social changes resulting from PPS in our rat model, providing a viable therapeutic target for the modulation of social behaviour following early-life stress [21].

We also report an overlap with DEGs following a social play paradigm [55], which used similar social protocols to those employed herein. Interestingly, we see that in PPS rats, genes that were increased with social play are decreased instead—further suggesting the way PPS rats are processing the social interaction is fundamentally different to control rats, which could result in the different behavioural phenotypes. It should be noted, however, that these gene sets were taken from different brain regions and different sexes to those employed in this study. Alugubelly et al. [55] suggest that increased inhibitory GPCR signalling is required after social play to regulate the reward and stress responses. Considering that we observe differences in GPCR signalling following a similar social play paradigm in PPS rats compared to CON rats, this suggests that this mechanism to restore the reward/stress systems to homoeostasis may be altered in our PPS rats.

Altered transmission, particularly monoaminergic, through GPCRs is consistently altered in models of early-life stress and it is hypothesised that dysfunction in these pathways can lead to the reprogramming of neurocircuitry that can enhance the risk of developing psychiatric and mood disorders in adulthood [78]. This evidence is particularly striking in the PFC, where a reduction in inhibitory Gi-coupled signalling and increased excitatory Gq-couple signalling is reported following ELS [78]. Our results add to this body of evidence to suggest that altered or dysfunctional GPCR signalling can act as a convergence point for models of early-life stress, however, more information is needed on the specific GPCRs that are altered and their effect on neurocircuitry to fully understand the molecular consequences of early trauma.

There are some limitations to the current study. For example, we used only female rats, and the stress response has been demonstrated to be variable between sexes [79, 80], as has the response to social interaction. Hence, further work is needed to compare the effect of sex on the response to PPS. Also, the sample size did not allow for the identification of low or high responders to PPS, which would be an important comparison to consider in the context of resilience vs vulnerability [81] following early-life stress. Furthermore, snap freezing the brain does not remove blood cells, so some of the signals may have arisen from cells such as leukocytes as well as a range of resident brain cells that are also potentially affected by PPS [82]. It would be interesting in future studies to analyse different cell populations in isolation, to determine the cellular origin of the differential expression profiles that arise following PPS.

To conclude, this study demonstrates a set of differentially expressed genes in the PFC of PPS rats directly following a social test. This highlights the importance of behavioural or stress challenges to activate neural paths altered by PPS, revealing the effects of PPS on gene expression. Future research should focus on the potential epigenomic basis of these gene expression changes and the effect of the same stressors and social test on different brain regions to identify the cellular mechanisms impacted by PPS in behaviourally relevant circuits. Such studies will be important for gaining a more complete and detailed insight into the biological mechanisms that underlie the risk of mental illness that emerges from experiencing stress before puberty—an understanding that is vital for more targeted interventions to help those individuals with experiences of early-life trauma.

## Supplementary information

Supplementary Table 1

Supplementary Table 2

Supplementary Table 3

Supplementary Table 4
